# A polymorphism rs4705341 in the flanking region of miR-143/145 predicts risk and prognosis of colorectal cancer

**DOI:** 10.18632/oncotarget.11387

**Published:** 2016-08-19

**Authors:** Ruifen Sun, Peng Chen, Lijuan Li, Hong Sun, Xinwen Nie, Yundan Liang, Fang Yuan, Yan Pu, Peng Bai, Lin Zhang, Linbo Gao

**Affiliations:** ^1^ Laboratory of Molecular and Translational Medicine, West China Institute of Women and Children's Health, Key Laboratory of Birth Defects and Related Diseases of Women and Children (Sichuan University), Ministry of Education, West China Second University Hospital, Sichuan University, Chengdu, Sichuan 610041, P.R. China; ^2^ Department of Immunology, West China School of Preclinical and Forensic Medicine, Sichuan University, Chengdu, Sichuan 610041, P.R. China; ^3^ Central Laboratory, Yunnan University of Chinese Traditional Medicine, Yunnan, Kunming 650500, P.R. China; ^4^ Department of Obstetrics and Gynaecology, West China Second University Hospital, Sichuan University, Chengdu, Sichuan 610041, P.R. China; ^5^ Department of Forensic Biology, West China School of Preclinical and Forensic Medicine, Sichuan University, Chengdu, Sichuan 610041, P.R. China

**Keywords:** miR-143/145, polymorphism, survival, colorectal cancer

## Abstract

The aim of this study was to investigate the effect of a polymorphism rs4705341 in the flanking region of miR-143/145 on the risk of colorectal cancer (CRC). The rs4705341 polymorphism was analyzed in 1002 cases and 1062 controls using a polymerase chain reaction-restriction fragment length polymorphism method. We found a significantly reduced CRC susceptibility with miR-143/145 rs4705341 in homozygote comparison (adjusted OR = 0.66, 95%CI, 0.50-0.88, *P* = 0.004), dominant genetic model (adjusted OR = 0.80, 95%CI, 0.67-0.96, *P* = 0.015), recessive genetic model (adjusted OR = 0.73, 95%CI, 0.56-0.94, *P* = 0.016), and allele comparison (adjusted OR = 0.83, 95%CI, 0.73-0.94, *P* = 0.004). Stratification analysis showed that the rs4705341 was related to differentiated status, clinical stage I-II, and patients without lymph node metastasis. Moreover, patients with rs4705341GG had a longer overall survival (adjusted HR = 5.57, 95%CI, 0.95-32.68). These findings indicate that the miR-143/145 rs4705341 may be used as a potential biomarker for the development and prognosis of CRC.

## INTRODUCTION

Colorectal cancer (CRC) takes almost 700,000 lives every year, making it one of the most common forms of neoplasia in industrial countries [1]. As the world developing, its incidence is increasing [2]. Even though the precise molecular mechanism of CRC has still remained unclear, it has been considered to be consequence of intrinsic genetic variants that are influenced by local environmental factors [3, 4]. With advances in the recognizing of the pathophysiology and genetics of CRC, genetic biomarkers were assumed to predict the future presence of cancer and even direct the approach to therapy [5].

MicroRNAs (miRNAs) are 19-25-nucleotides non-coding RNA molecules that can result in the degradation or repression of mRNA by sequence-specific base pairing on the 3′-untranslated regions of target mRNA [6, 7]. Recent studies have identified that genetic polymorphisms in the flanking region of miRNAs were associated with cancer susceptibility [8–24]. MiR-143 and miR-145, extensively studied miRNAs, are highly expressed in mesenchymal cells, with the function of inducing cellular differentiation [25, 26]. In our previous work, we found an rs4705341 polymorphism in the flanking region of miR-143/145 (−2545bp upstream from miR-143/145) was associated with CRC risk [14]. However, the sample size is very limited. In this study, we collected more samples to confirm the result and analyzed the relationship of the rs4705341 polymorphism and outcome of CRC patients.

## RESULTS

### Characteristics of the study population

We conducted a large-scale case-control study including 1002 CRC patients and 1062 healthy controls. The mean age of cases and controls was 60.7 and 57.9 years, respectively. The male/female ratio was about 3:2 in both case and control group. Among the cases, 564 (56.3%) were well-moderately differentiated CRC and 438 (43.7%) were poorly differentiated CRC. Five hundred eighty-four (58.3%) were stage I–II, and 418 (41.7%) were stage III–IV. Most of the cases (64.7%) had no lymph node metastasis (Table 1).

**Table 1:** Characteristics of the study population

Variables	Controls (n = 1062)	Patients with CRC (n = 1002)
Age (years, Mean ± SD)	57.9 (± 10.1)	60.7 (± 13.5)
Gender (%)		
Male	623 (58.7)	615 (61.4)
Female	439 (41.3)	387 (38.6)
Differentiated status (%)		
Well-Moderately		564 (56.3)
Poorly-Undifferentiated		438 (43.7)
Clinical stage (%)		
I- II		584 (58.3)
III- IV		418 (41.7)
Lymph node metastasis (%)		
Yes		354 (35.3)
No		648 (64.7)

CRC, colorectal cancer

SD, standard deviation

### Large-scale study confirms the association of the rs4705341 polymorphism with CRC risk

The distribution of the rs4705341 polymorphism was consistent with the Hardy-Weinberg equilibrium in the case and control group (*P* = 0.09 and 0.22), indicating that we obtained representative samples of the general population. As shown in Table 2, significantly reduced CRC risk was found to be associated with GG genotype and G allele when compared with AA genotype and A allele (GG vs. AA: adjusted OR = 0.66, 95%CI, 0.50-0.88, *P* = 0.004; G vs. A: adjusted OR = 0.83, 95%CI, 0.73-0.94, *P* = 0.004). Similarly reduced risk was also observed in a dominant model (adjusted OR = 0.80, 95%CI, 0.67-0.96, *P* = 0.015) and recessive model (adjusted OR = 0.73, 95%CI, 0.56-0.94, *P* = 0.016).

**Table 2:** Association between the rs4705341 polymorphism in the flanking region of miR-143/145 and risk of CRC

Polymorphism	Controls (n=1062) (%)	CRC (n=1002) (%)	Logistic Regression (Crude)		Logistic Regression (Adjusted) †	
			OR (95%CI)	*P* value	OR (95%CI)	*P* value
AA	381 (35.9)	412 (41.1)	1.00 (Ref)		1.00 (Ref)	
AG	526 (49.5)	480 (47.9)	0.84 (0.70-1.02)	0.07	0.84 (0.70-1.02)	0.07
GG	155 (14.6)	110 (11.0)	0.66 (0.50-0.87)	0.003	0.66 (0.50-0.88)	0.004
Dominant model			0.80 (0.67-0.96)	0.014	0.80 (0.67-0.96)	0.015
Recessive model			0.72 (0.56-0.94)	0.014	0.73 (0.56-0.94)	0.016
A allele	1288 (60.6)	1304 (65.1)	1.00 (Ref)		1.00 (Ref)	
G allele	836 (39.4)	700 (34.9)	0.83 (0.73-0.94)	0.003	0.83 (0.73-0.94)	0.004

CRC, colorectal cancer

OR, odds ratio

CI, confidence interval

† OR was adjusted by age and gender.

Next, we divided the patients into 2 groups by differentiated status, clinical stage and lymph node metastasis. We found a significantly reduced risk in either well-moderately CRC patients (GG vs. AA: adjusted OR = 0.66, 95%CI, 0.47-0.92, *P* = 0.014; G vs. A: adjusted OR = 0.82, 95%CI, 0.71-0.96, *P* = 0.011; dominant model: adjusted OR = 0.79, 95%CI, 0.64-0.97, *P* = 0.03) or poorly-undifferentiated CRC patients (GG vs. AA: adjusted OR = 0.66, 95%CI, 0.45-0.96, *P* = 0.03; G vs. A: adjusted OR = 0.83, 95%CI, 0.70-0.98, *P* = 0.03). The protective effect was also found in patients with clinical stage I-II (GG vs. AA: adjusted OR = 0.60, 95%CI, 0.43-0.85, *P* = 0.004; G vs. A: adjusted OR = 0.80, 95%CI, 0.68-0.93, *P* = 0.003; dominant model: adjusted OR = 0.76, 95%CI, 0.62-0.94, *P* = 0.012; recessive model: adjusted OR = 0.68, 95%CI, 0.49-0.94, *P* = 0.016) but not in patients with clinical stage III-IV. Moreover, the frequencies of GG genotype and G allele were significantly different in patients without lymph node metastasis (GG vs. AA: adjusted OR = 0.61, 95%CI, 0.44-0.84, *P* = 0.002; G vs. A: adjusted OR = 0.80, 95%CI, 0.69-0.93, *P* = 0.003; dominant model: adjusted OR = 0.77, 95%CI, 0.63-0.94, *P* = 0.012; recessive model: adjusted OR = 0.69, 95%CI, 0.51-0.94, *P* = 0.018) rather than in patients with lymph node metastasis (Table 3).

**Table 3:** Stratified analyses of the rs4705341 polymorphism with clinical features of CRC

Clinical features	CRC patients (%)		Controls (%)	Ca I vs. Controls		Ca II vs. Controls	
	Ca I	Ca II		Adjusted OR (95%CI) †	*P* value	Adjusted OR (95%CI) †	*P* value
Differentiated status	Well–moderately	Poorly–undifferentiated					
AA	232 (41.1)	180 (41.1)	381 (35.9)	1.00 (Ref)		1.00 (Ref)	
AG	269 (47.7)	211 (48.2)	526 (49.5)	0.82 (0.66-1.03)	0.09	0.85 (0.67-1.09)	0.20
GG	63 (11.2)	47 (10.7)	155 (14.6)	0.66 (0.47-0.92)	0.014	0.66 (0.45-0.96)	0.03
Dominant model				0.79 (0.64-0.97)	0.03	0.80 (0.64-1.02)	0.07
Recessive model				0.73 (0.53-1.00)	0.05	0.71 (0.50-1.01)	0.05
A allele	733 (65.0)	571 (65.2)	1288 (60.6)	1.00 (Ref)		1.00 (Ref)	
G allele	395 (35.0)	305 (34.8)	836 (39.4)	0.82 (0.71-0.96)	0.011	0.83 (0.70-0.98)	0.03
Clinical stages	I-II	III-IV					
AA	248 (42.5)	164 (39.2)	381 (35.9)	1.00 (Ref)		1.00 (Ref)	
AG	276 (47.3)	204 (48.8)	526 (49.5)	0.81 (0.65-1.01)	0.06	0.89 (0.70-1.14)	0.37
GG	60 (10.3)	50 (12.0)	155 (14.6)	0.60 (0.43-0.85)	0.004	0.75 (0.52-1.08)	0.11
Dominant model				0.76 (0.62-0.94)	0.012	0.86 (0.68-1.09)	0.21
Recessive model				0.68 (0.49-0.94)	0.016	0.80 (0.57-1.12)	0.19
A allele	772 (66.1)	532 (63.6)	1288 (60.6)	1.00 (Ref)		1.00 (Ref)	
G allele	396 (33.9)	304 (36.4)	836 (39.4)	0.80 (0.68-0.93)	0.003	0.88 (0.74-1.04)	0.12
Lymph node metastasis	No	Yes					
AA	274 (42.3)	138 (39.0)	381 (35.9)	1.00 (Ref)		1.00 (Ref)	
AG	306 (47.2)	174 (49.1)	526 (49.5)	0.81 (0.66-1.01)	0.06	0.91 (0.70-1.18)	0.47
GG	68 (10.5)	42 (11.9)	155 (14.6)	0.61 (0.44-0.84)	0.002	0.75 (0.50-1.11)	0.14
Dominant model				0.77 (0.63-0.94)	0.012	0.87 (0.68-1.12)	0.28
Recessive model				0.69 (0.51-0.94)	0.018	0.79 (0.55-1.14)	0.20
A allele	854 (65.9)	450 (63.6)	1288 (60.6)	1.00 (Ref)		1.00 (Ref)	
G allele	442 (34.1)	258 (36.4)	836 (39.4)	0.80 (0.69-0.93)	0.003	0.87 (0.73-1.04)	0.12

CRC, colorectal cancer

Ca I, group I of cases; Ca II, group II of cases

OR, odds ratio

CI, confidence interval

† OR was adjusted by age and gender.

### The rs4705341GG genotype exhibited a better overall survival

**Figure 1:**
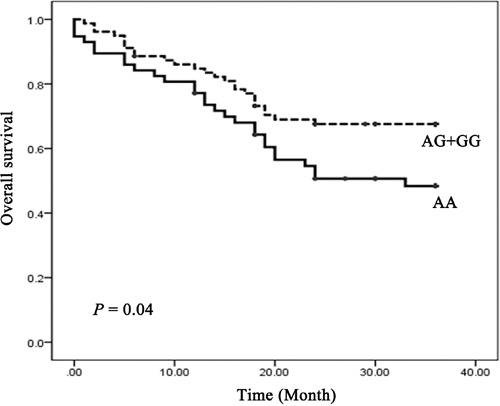
Kaplan-Meier overall survival curve for CRC patients by rs4705341 genotypes

Log-rank test and Cox proportional hazards model were performed to examine the association of the rs4705341 with 3-year overall survival of CRC. The mean survival time in AA carriers was 22.7 months, whereas the mean survival time in AG/GG carriers was 26.6 months. Significant difference was observed between the 2 groups with a *P* value of 0.04 (Figure 1). As shown in Table 4, the rs4705341GG genotype was significantly associated with a better survival of CRC (adjusted HR = 5.57, 95%CI, 0.95-32.68, *P* = 0.038).

**Table 4:** Association between the rs4705341 polymorphism and patient's survival

Polymorphism	Total (n=115)	Death (n=53)	Adjusted HR (95%CI) †	*P* value
AA	50	28	1.00 (Ref)	
AG	55	23	1.57 (0.68-3.60)	0.29
GG	10	2	5.57 (0.95-32.68)	0.038
Dominant model			1.86 (0.84-4.10)	0.12
Recessive model			4.54 (0.82-25.03)	0.06

HR, hazard ratio

CI, confidence interval

† HR was adjusted by age and gender.

## DISCUSSION

In this study, we confirmed an association of the rs4705341 polymorphism in the flanking region of miR-143/145 with the risk of CRC in a Chinese population, with the rs4705341G allele having a 0.83-fold decreased risk of CRC. Stratification analysis revealed that the rs4705341 was related to clinical features of CRC patients. These findings indicate that the rs4705341 polymorphism may be involved in the etiology and prognosis of CRC.

Recently, genetic variants in the flanking region of miRNA may contribute to individual's susceptibility to cancer [8–24]. An rs999885 in the promoter region of miR-106b-25 cluster had an increased risk and a protective effect on the prognosis of intermediate or advanced hepatocellular carcinoma [10, 15]. An rs10877887 in the promoter of let-7 family had an elevated risk of lung cancer [18] and an increased death risk of hepatocellular carcinoma [11] but a reduced risk of papillary thyroid carcinoma [16]. An rs4938723 in the promoter of pri-miR-34b/c had a decreased risk of colorectal cancer [12] and gastric cancer [19, 20], but an increased risk of hepatocellular carcinoma [13, 23], renal cell cancer [21], and nasopharyngeal carcinoma [24]. Based on this background, we hypothesized that polymorphism in the flanking region of miR-143/145 may be related to cancer risk. This hypothesis was confirmed by our previous work [8, 14] and findings from Chu et al. who found an rs4705342T>C in the flanking region of miR-143 decreasing the risk of prostate cancer and the risk-associated T allele increasing the protein-binding affinity and reducing the luciferase activity [9]. In this study, we focused on a novel polymorphism rs4705341 in the flanking region of miR-143/145 and found that the rs4705341G allele can protect against not only the development of CRC but also clinical outcome.

Several limitations should be discussed. CRC is a complex disease with multi-factors rather than a single factor involved. In this study, only genetic factor was taken into consideration. Further association studies investigating gene-gene and gene-environment interaction are of great importance to clarify the pathogenesis of CRC. Distribution of the rs4705341 is different in diverse ethnic groups. Only Chinese Han subjects were enrolled in this study. Therefore, the results cannot be directly extended to other ethnicities. Further investigations are warranted to validate the results in different races. Although we found that the rs4705341 polymorphism is potentially functional, the convincing mechanism is not fully provided. Further analysis of exact mechanism is needed.

In conclusion, we reported for the first time that the rs4705341 GG genotype was associated with a decreased CRC susceptibility and better outcome, suggesting that the miR-143/145 rs4705341 may be a potential biomarker for the development and prognosis of CRC.

## MATERIALS AND METHODS

### Study subjects and samples

CRC patients were consecutively recruited from the West China Hospital of Sichuan University, the Luoyang Central Hospital Affiliated to Zhengzhou University, and the Affiliated Hospital of North Sichuan Medical College. Eight hundred and sixty-five paraffin-embedded tissue specimens (10 μm thickness) and 256 peripheral venous blood samples from CRC patients were collected between January 2010 and February 2015. The diagnosis of CRC was determined by histopathological analysis. Clinical information, including age, gender, differentiated status, clinical stage and lymph node metastasis, was retrieved from surgical and pathological records and summarized in Table 1. Follow-up data was obtained from telephone calls. During the same period, 1124 healthy volunteers visiting the hospital for physical examination was selected as controls. Individuals who had a self-reported history of colorectal disease or antecedents of malignancy were excluded from the control group. All the participants were unrelated Han population living in the central and western area of China. The study was conducted with the approval of the ethics committee of the hospital. All the subjects agreed to participant in the study.

### DNA extraction

Genomic DNA from EDTA-anticogulated peripheral blood was extracted by using a commercial extraction kit (Bioteke, Beijing, China) and DNA from paraffin-embedded tissue sections was isolated by using a Puregene® DNA isolated kit (Qiagen, Hilden, Germany) according to the instruction manual.

### Genotyping

The rs4705341 polymorphism was analyzed using a polymerase chain reaction-restriction fragment length polymorphism method (PCR-RFLP). The primer sequences were 5′-CCTCGGCTTCCCAAAGTGATC-3′ (forward) and 5′-TCCCAGCCAGATAACTGCTTTCC-3′ (reverse). PCR reaction was performed in a total volume of 10 μl, containing 50 ng genomic DNA, 20 pM each primer, and 5 μl 2X PCR mix (Aidlab Biotech, Beijing, China). The PCR products were digested with *Bcl* I (New England BioLabs, Beverly, MA). The rs4705341A allele was cut into two fragments of 106 and 17 bp, whereas the rs4705341G allele remained intact. For quality control, the laboratory staff performing the genotyping analysis did not know the subjects' case-control status. Two research personnel independently read the gel pictures. The samples were reanalyzed if an agreement was not reached. Moreover, the genotyping method of PCR-RFLP was confirmed by sequencing (TsingKe, Chengdu, China) in 10% of all samples, and the results were all consistent. Due to DNA quality and quantity, 1062 controls and 1002 cases were genotyped successfully.

### Statistical analysis

All the analyses were done using SPSS software version 13.0 (SPSS Inc, Chicago, IL). Hardy-Weinberg equilibrium was measured by χ^2^ test. The χ^2^ test, with odds ratio (OR) and respective 95% confidence interval (CI), were applied to evaluate the differences of genotype and allele distribution among cases and controls. Genotypic association tests were determined using SNPstats [27], assuming heterozygous comparison, homozygote comparison, allele comparison, dominant, and recessive genetic models. Unconditional logistic regression was used to estimate adjusted OR based on age and gender. The association between the rs4705341 polymorphism with overall survival was analyzed using Kaplan-Meier plots and the log-rank test. Cox regression analysis was performed to estimate hazard ratio (HR) and 95%CI when controlling for age and gender. A *P* level of < 0.05 was considered as statistically significant.
